# A Theoretical Analysis of the Coherence-Induced Spectral Shift Experiments of Kandpal, Vaishya, and Joshi

**DOI:** 10.6028/jres.099.023

**Published:** 1994

**Authors:** John T. Foley, M. Wang

**Affiliations:** Department of Physics and Astronomy, Mississippi State University, Mississippi State, MS 39762

**Keywords:** correlation-Induced spectral shifts, optical coherence theory, radiomctry, spectroscopy, Wolf shifts

## Abstract

The optical system used by Kandpal, Vaishya, and Joshi in their experiments on coherence-induced spectral shifts is analyzed theoretically. An approximate form for the cross-spectral density in the secondary source plane is obtained, and it is shown that, contrary to the assertions of Kandpal, Vaishya, and Joshi, the corresponding complex degree of spectral coherence in this plane is wavelength dependent. After making some assumptions about the behavior of the interference filter used in the system, an approximate form for the spectrum of the light on-axis in the observation plane is obtained. It is shown that the peak wavelengths of this spectrum do not agree with those reported by Kandpal, Vaishya, and Joshi. Possible reasons for this disagreement arc discussed.

## 1. Introduction

In 1989 Kandpal, Vaishya, and Joshi of the National Physical Laboratory (India) published results of experiments in which they observed spectral shifts caused by a simple optical system [1]. In their experiments light from the exit aperture of an integrating sphere was imaged by a two lens system which had an interference filter between the lenses. They found that when a small aperture was placed in the image plane, the peak wavelength of the spectrum of the light measured on-axis in the far zone of the aperture was shifted away from the peak wavelength which occurred when no aperture was used.

They interpreted these shifts as being “Wolf shifts,” i.e., coherence-induced shifts of the type predicted by Wolf [2–3]. Their explanation [4] for the occurrence of the shifts was based on their assertion (which was neither proven directly experimentally nor justified theoretically) that the filter-lens combination eliminated the wavelength dependence of the complex degree of spectral coherence of the light in the image plane. If this assertion is true, then a coherence-induced change of the spectrum is to be expected when the light propagates from the image plane to the far zone, as was shown experimentally by Morris and Faklis [5] and Faklis and Morris [6]. Kandpal et al. [4] then argued that the introduction of a circular aperture in the image plane helped in modifying the spectrum in the far zone.

In this paper, we will analyze theoretically the optical system used in Ref. [1]. Many details of the system which did not appear in Ref. [1] were provided to us by the group at NPL [7]. The basic outline of our paper is as follows. In Sec. 2 the optical system is described, and the basic assumptions to be made throughout the paper are stated. In Sec. 3 an approximate form for the cross-spectral density of the light in the image plane is obtained, and it is shown that the corresponding complex degree of spectral coherence is *not* wavelength independent. In Sec. 4 the spectrum of the light on-axis in the observation plane is investigated, and an approximate form for it is obtained, both for the case in which no aperture is used, and for the case in which the small aperture is used. It is shown that, for each interference filter used, the peak wavelength in the latter case is shifted with respect to the peak wavelength in the former case. However, these shifts do not agree with those observed by the group at NPL. The shifts predicted by our analysis are much smaller, so small as to be unobservable to within the accuracy of their experiments. In Sec. 5 our conclusions are presented.

## 2. Optical System, Notation and Assumptions

**Fig. 1:**
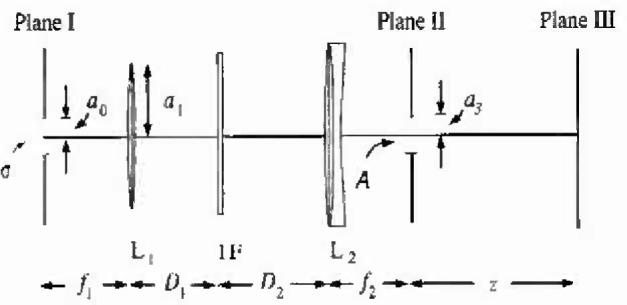
The optical system.

The optical system used by the group at NPL is pictured in Fig. 1. Just to the left of plane I there is an integrating sphere of radius 25 cm which has a 450 W tungsten halogen lamp at its center. Plane I contains the exit aperture, *σ*, of the integrating sphere. σ is a circular aperture of radius *a*_0_ = 0.12 cm, and it is in the front focal plane of a lens, L_1_, whose focal length is *f*_1_ = 5 cm and radius is *a*_1_ =0.45 cm.

There is an interference filter, IF, a distance *D*_1_ =20 cm behind L_1_, and a second lens, L_2_, a distance *D*_2_ = 30 cm behind the interference filter. L_2_ is an achromatic doublet, and its focal length is *f*_2_ = 20 cm. The radius of L_2_ and the transverse dimensions of the interference filter are all larger than *a*_1_. It is therefore a straightforward matter to show that L_1_ is the aperture stop for the system. In their experiments the group at NPL used six different interference filters; the shortest peak transmission wavelength used was 422 nm and the longest was 652 nm.

The exit aperture of the integrating sphere is imaged onto plane II. It is a straightforward matter to show that the magnification, *M*, for this imaging is:

$$M=-f_{2}/f_{1}=-4.$$ (1)

Two different kinds of things were done as regards plane II. In one case an aperture A of radius *a*_3_ was placed in plane II, and experiments were done for the values *a*_3_ =0.012 cm and 0.50 cm.^1^ In the second case no aperture was used. For notational convenience, we will treat the latter case as if an aperture A of radius *a*_3_ = ∞ were in plane II. Also, since no shift was observed in the 0.50 cm aperture case, we will not consider that case. Plane III, where the measurements were made, is a distance *z* = 100 cm from plane II.

The following notation will be used throughout this paper. The locations with respect to the optical axis of points in the planes I, II, and III will be specified by, respectively, the two-dimensional position vectors *ρ″, ρ*′, and *ρ*. The locations of points in the planes occupied by L_1_ and L_2_ will be specified by, respectively, the two-dimensional position vectors *α*_1_ and *α*_2_, The lengths of vectors will be denoted by the corresponding non-boldfaced symbols, e.g., *ρ″=|ρ″|.*

The following assumptions will be made. As concerns the properties of the light coming from the integrating sphere, it will be assumed that the exit aperture of the integrating sphere radiates as a uniform lambertian source. Let the spectral radiance of this source, at angular frequency *ω*, be denoted by.*B*_0_(*ω*). The cross-spectral density, $W^{\left(1\right)}\left(\rho_{1}^{\prime \prime },\rho_{2}^{\prime \prime },\omega \right)$ of the light in plane I can be written as [9–11]:^2^

$$\begin{array}{l} \;W^{\left(1\right)}\left(\rho_{1}^{\prime \prime },\rho_{2}^{\prime \prime },\omega \right)=2\pi B_{0}\left(\omega \right)\\ \times j_{0}\left(k\left|\rho_{2}^{\prime \prime }-\rho_{1}^{\prime \prime }\right|\right)P_{0}\left(\rho_{1}^{\prime \prime }\right)P_{0}\left(\rho_{2}^{\prime \prime }\right), \end{array}$$ (2)

where *k* is the wavenumber of the light,

k=ω/c=2π/λ, (3)

*j*_0_ is the spherical Bessel function of the first kind of order zero,

$$j_{0}\left(u\right)=\frac{\sin\left(u\right)}{u},$$ (4)

and *P*_0_ is the pupil function for the exit aperture of the integrating sphere,

$$P_{0}\left(\rho^{\prime \prime }\right)=\text{circ}\left(\rho^{\prime \prime }/a_{0}\right).$$ (5)

In Eq. (3)*c* is the speed of light in vacuo and λ is the wavelength of the light. In Eq. (5) circ is the circle function,

$$\begin{array}{r} \text{circ}\left(u\right)=1,\text{if}\;\left|u\right|\leq 1,\\ =0,\text{if}\;\left|u\right|>1. \end{array}$$ (6)

Secondly, since L_1_ is the aperture stop for the system, it will be assumed that the finiteness of the transverse sizes of L_2_ and the interference filter can be neglected. Thirdly, it will be assumed that the transmittance of the interference filter may be described by the Lissberger-Wilcock model [12–13], and that its behavior is such that the approximation described in Sec. 4.1 is valid. In addition, both lenses will be treated as thin lenses, and the possibility of chromatic aberration introduced by either L_1_ or L_2_ will not be taken into account. The paraxial approximation will be used throughout our work.

## 3. Cross-Spectral Density of the Light in Plane II

In this section, the cross-spectral density in plane II will be investigated. First the coherent impulse response function for the propagation from plane I to plane II will be determined. This result will then be used to obtain an “exact” (within the paraxial approximation) expression for the cross-spectral density incident upon plane II. Approximations appropriate to the NPL experiments will then be used to obtain an approximate expression for the cross-spectral density in plane II. The corresponding spectrum and complex degree of spectral coherence in plane II will then be calculated and discussed.

### 3.1 Coherent Impulse Response Function for the Propagation from Plane I to Plane II

Let *P″* be a point in plane I located by position vector *ρ″*, and let P′ be a point in plane II located by position vector *ρ′.* The coherent impulse response function, *h*(*ρ′, ρ″, ω*), for the propagation from plane I to plane II is the field at P′ due to a monochromatic, unit amplitude point source of angular frequency *ω* at P″. In order to calculate this function we must first discuss the effect of the interference filter.

**Fig. 2:**
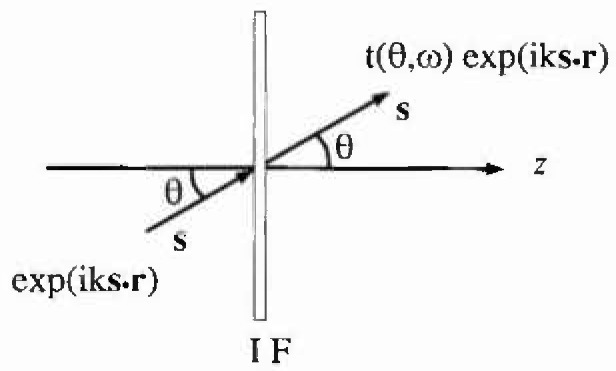
The effect of the interference filter on a plane wave.

The effect of the interference filter on a plane wave is depicted in Fig. 2. Here a unit amplitude, monochromatic plane wave of angular frequency *ω* propagating in the direction specified by the unit vector *s* is incident upon the filter at an angle of incidence *θ.* If, as we have assumed, the finiteness of the transverse size of the filter can be neglected, the effect of the interference Filter is to change the amplitude of the plane wave by the factor *t*(*θ, ω*), the amplitude transmission function of the interference filter.

When this effect is taken into account, it can be shown (see Appendix A) that in the paraxial approximation *h*(*ρ′, ρ″, ω*) is the product of *t*(*ρ′*/*f*_2_, *ω*), the amplitude transmission function of the interference filter evaluated at *θ=ρ′*/*f*_2_, and *h_N_*(*ρ′, ρ″, ω*), the coherent impulse response function (paraxial form) for the case when there is no interference filter present, i.e.,

$$h\left(\rho^{\prime },\rho^{\prime \prime },\omega \right)=t\left(\rho^{\prime }/f_{2},\omega \right)h_{N}\left(\rho^{\prime },\rho^{\prime \prime },\omega \right),$$ (7)

where

$$\begin{array}{c} h_{N}\left(\rho^{\prime },\rho^{\prime \prime },\omega \right)=C\left(\omega \right)\exp\left\{ik\left[\phi \left(\rho^{\prime \prime }\right)+x\left(\rho^{\prime }\right)\right]\right\}\\ \times \text{Besinc}\left(\frac{ka_{1}}{f_{1}}\left|\rho^{\prime \prime }-\frac{1}{M}\rho^{\prime }\right|\right) \end{array}$$ (8)

and

$$C\left(\omega \right)=\frac{1}{M}\frac{\pi a_{1}^{2}}{\lambda^{2}f_{1}^{2}}$$ (9)

$$\phi \left(\rho^{\prime \prime }\right)=f_{1}+\left({\rho^{\prime \prime }}^{2}/2f_{1}\right),$$ (10)

$$x\left(\rho^{\prime }\right)=f_{2}+D+\left(1-\frac{D}{f_{2}}\right)\left({\rho^{\prime }}^{2}/2f_{2}\right),$$ (11)

$$\text{Besinc}\left(u\right)=2J_{1}\left(u\right)/u.$$ (12)

In Eq. (11)*D* is the distance from L_1_ to L_2_, i.e., *D = D*_1_ *+D*_2_. In Eq. (12)*J*_1_ is the Bessel function of the first kind of order one.

### 3.2 Cross-Spectral Density of the Light Incident Upon Plane II

The cross-spectral density of the light incident upon plane II, $W^{\left(\mathrm{II}-\right)}\left(\rho_{1}^{\prime },\rho_{2}^{\prime },\omega \right)$, is related to the cross-spectral density in the plane I by the expression [14]

$$\begin{array}{c} W^{\left(\mathrm{II}-\right)}\left(\rho_{1}^{\prime },\rho_{2}^{\prime },\omega \right)=\int_{\sigma }\int_{\sigma }h*\left(\rho_{1}^{\prime },\rho_{1}^{\prime \prime },\omega \right)h\left(\rho_{2}^{\prime },\rho_{2}^{\prime \prime },\omega \right)\\ \times W^{\left(I\right)}\left(\rho_{1}^{\prime \prime },\rho_{2}^{\prime \prime },\omega \right)d^{2}\rho_{1}^{\prime \prime }d^{2}\rho_{2}^{\prime \prime }, \end{array}$$ (13)

where *h*(*ρ′, ρ″, ω*) is the coherent impulse response function for the propagation from plane I to plane II. Upon substituting Eqs. (2) and (7) into Eq. (13), and using Eq. (8) in the result, we find that

$$\begin{array}{c} W^{\left(\mathrm{II}-\right)}\left(\rho_{1}^{\prime },\rho_{2}^{\prime },\omega \right)=2\pi B_{0}\left(\omega \right)t*\left(\rho_{1}^{\prime }/f_{2},\omega \right)t\left(\rho_{2}^{\prime }/f_{2},\omega \right)\\ \times F\left(\rho_{1}^{\prime },\rho_{2}^{\prime },\omega \right), \end{array}$$ (14)

where

$$\begin{array}{c} F\left(\rho_{1}^{\prime },\rho_{2}^{\prime },\omega \right)=C^{2}\left(\omega \right)E\left(\rho_{1}^{\prime },\rho_{2}^{\prime \prime },\omega \right)\\ \times \int_{\sigma }\int_{\sigma }\bar{E}\left(\rho_{1}^{\prime \prime },\rho_{2}^{\prime \prime },\omega \right)\;\text{Besinc}\left(\frac{ka_{1}}{f_{1}}\left|\rho_{1}^{\prime \prime }-\frac{1}{M}\;\rho_{1}^{\prime }\right|\right)\\ \times \text{Besinc}\left(\frac{ka_{1}}{f_{1}}\left|\rho_{2}^{\prime \prime }-\frac{1}{M}\rho_{2}^{\prime }\right|\right)j_{0}\left(k\left|\rho_{2}^{\prime \prime }-\rho_{1}^{\prime \prime }\right|\right)\\ \times d^{2}\rho_{1}^{\prime \prime }d^{2}\rho_{2}^{\prime \prime }, \end{array}$$ (15)

and $E\left(\rho_{1}^{\prime },\rho_{1}^{\prime },\omega \right)$ and $\bar{E}\left(\rho_{1}^{\prime \prime },\rho_{2}^{\prime \prime },\omega \right)$ are phase factors:

$$E\left(\rho_{1}^{\prime },\rho_{2}^{\prime },\omega \right)=\exp\left[\mathrm{ik}\left(1-\frac{D}{f_{2}}\right)\left(\rho_{2}^{\prime 2}-\rho_{1}^{\prime 2}\right)/2f_{2}\right],$$ (16)

$$\bar{E}\left(\rho_{1}^{\prime \prime },\rho_{2}^{\prime \prime },\omega \right)=\exp\left[\mathrm{ik}\left(\rho_{2}^{\prime \prime 2}-\rho_{1}^{\prime \prime 2}\right)/2f_{1}\right].$$ (17)

Equation (14), with $F\left(\rho_{1}^{\prime },\rho_{2}^{\prime },\omega \right)$ given by Eq. (15), is an exact (within the paraxial approximation) expression for the cross-spectral density incident upon plane II. However, the integral on the right hand side of Eq. (15) cannot be evaluated analytically. Nevertheless, for the NPL system certain approximations are appropriate which simplify $F\left(\rho_{1}^{\prime },\rho_{2}^{\prime },\omega \right)$ considerably.

First, it is shown in Appendix B that for the NPL parameter values we may set $\bar{E}\left(\rho_{1}^{\prime \prime },\rho_{2}^{\prime \prime },\omega \right)\approx 1$ in the integrand on the right hand side of Eq. (15). Hence, to a good approximation

$$\begin{array}{c} F\left(\rho_{1}^{\prime },\rho_{2}^{\prime },\omega \right)=C^{2}\left(\omega \right)E\left(\rho_{1}^{\prime },\rho_{2}^{\prime },\omega \right)\int_{\sigma }\int_{\sigma }\times \text{Besinc}\left(\frac{ka_{1}}{f_{1}}\left|\rho_{1}^{\prime \prime }-\frac{1}{M}\rho_{1}^{\prime }\right|\right)\text{Besinc}\left(\frac{ka_{1}}{f_{1}}\left|\rho_{2}^{\prime \prime }-\frac{1}{M}\rho_{2}^{\prime }\right|\right)\\ \times j_{0}\left(k\left|\rho_{2}^{\prime \prime }-\rho_{1}^{\prime \prime }\right|\right)d^{2}\rho_{1}^{\prime \prime }d^{2}\rho_{2}^{\prime \prime }. \end{array}$$ (18)

Next, it is shown in Appendix C that for the NPL parameter values,

$$\begin{array}{c} \int_{\sigma }\int_{\sigma }\text{Besinc}\;\left(\frac{ka_{1}}{f_{1}}\left|\rho_{1}^{\prime \prime }-\frac{1}{M}\rho_{1}^{\prime }\right|\right)\\ \times \text{Besinc}\;\left(\frac{ka_{1}}{f_{1}}\left|\rho_{2}^{\prime \prime }-\frac{1}{M}\rho_{2}^{\prime }\right|\right)j_{0}\left(k\left|\rho_{2}^{\prime \prime }-\rho_{1}^{\prime \prime }\right|\right)d^{2}\rho_{1}^{\prime \prime }d^{2}\rho_{2}^{\prime \prime }\\ \approx \frac{1}{2C^{2}\left(\omega \right)}{\left(\frac{a_{1}}{f_{2}}\right)}^{2}\text{Besinc}\;\left(\frac{ka_{1}}{f_{2}}\left|\rho_{2}^{\prime }-\rho_{1}^{\prime }\right|\right)\\ \times \text{circ}\;\left(\rho_{1}^{\prime }/\left|M\right|a_{0}\right)\text{circ}\;\left(\rho_{2}^{\prime }/\left|M\right|a_{0}\right). \end{array}$$ (19)

It then follows from Eqs. (14), (18), and (19) that the cross-spectral density of the light incident upon plane II is given, to a good approximation, by the expression

$$\begin{array}{c} W^{\left(\mathrm{II}\right)}\left(\rho_{1}^{\prime },\rho_{2}^{\prime },\omega \right)=\frac{\pi a_{1}^{2}}{f_{2}^{2}}B_{0}\left(\omega \right)t*\left(\rho_{1}^{\prime }/f_{2},\omega \right)\\ \times t\left(\rho_{2}^{\prime }/f_{2},\omega \right)E\left(\rho_{1}^{\prime },\rho_{2}^{\prime },\omega \right)\text{Besinc}\;\left(\frac{ka_{1}}{f_{2}}\left|\rho_{2}^{\prime }-\rho_{1}^{\prime }\right|\right)\\ \times \text{circ}\left(\rho_{1}^{\prime }/\left|M\right|a_{0}\right)\text{circ}\left(\rho_{2}^{\prime }/\left|M\right|a_{0}\right). \end{array}$$ (20)

The spectrum, *S*^(II−)^ (*ρ′, ω*), of the light incident upon plane II at the point P′ located by the vector *ρ′* and at the angular frequency *ω* is obtained from the corresponding cross-spectral density by setting $\rho_{1}^{\prime }=\rho_{2}^{\prime }=\rho^{\prime }$.^3^ It therefore follows from Eq. (20) that

$$S^{\left(\mathrm{II}-\right)}\left(\rho^{\prime },\omega \right)=\frac{\pi a_{1}^{2}}{f_{2}^{2}}B_{0}\left(\omega \right){\left|t\left(\rho^{\prime }/f_{2},\omega \right)\right|}^{2}\text{circ}\left(\rho^{\prime }/\left|M\right|a_{0}\right).$$ (21)

It can be seen from this expression that this spectrum is nonuniform (since *t* depends on *ρ′*) and occupies the region *ρ′*≤|*M*|*a*_0_, i.e., the region which corresponds to the geometrical optics image of *σ*, the exit aperture of the integrating sphere.

### 3.3 Cross-Spectral Density, Spectrum, and Complex Degree of Spectral Coherence in Plane II

The cross-spectral density, $W^{\left(\mathrm{II}\right)}\left(\rho_{1}^{\prime },\rho_{2}^{\prime },\omega \right)$ in plane II (with the effect of the aperture A taken into account) is given by the expression

$$W^{\left(\mathrm{II}\right)}\left(\rho_{1}^{\prime },\rho_{2}^{\prime },\omega \right)=W^{\left(\mathrm{II}-\right)}\left(\rho_{1}^{\prime },\rho_{2}^{\prime },\omega \right)\text{circ}\left(\rho_{1}^{\prime }/a_{3}\right)\text{circ}\left(\rho_{2}^{\prime }/a_{3}\right).$$ (22)

Upon substituting the approximate Eq. (20) into Eq. (22), we find that the corresponding approximate expression for the cross-spectral density of the light in plane II is

$$\begin{array}{c} W^{\left(\mathrm{II}\right)}\left(\rho_{1}^{\prime },\rho_{2}^{\prime },\omega \right)=\frac{\pi a_{1}^{2}}{f_{2}^{2}}B_{0}\left(\omega \right)t*\left(\rho_{1}^{\prime }/f_{2},\omega \right)\\ \times t\left(\rho_{2}^{\prime }/f_{2},\omega \right)E\left(\rho_{1}^{\prime },\rho_{2}^{\prime },\omega \right)\text{Besinc}\left(\frac{ka_{1}}{f_{2}}\left|\rho_{2}^{\prime }-\rho_{1}^{\prime }\right|\right)\\ \times \text{circ}\left(\rho_{1}^{\prime }/a\right)\text{circ}\left(\rho_{2}^{\prime }/a\right), \end{array}$$ (23)

where *a* is the smaller of |*M*|*a*_0_, and *a*_3_, i.e., *a* =min(|*M*|*a*_0_, *a*_3_).

The physical significance of the parameter *a* is that it is the radius of the “secondary source.” If *a*_3_ *< |M|a*_0_ part of the incident light is blocked by the aperture A, and therefore the radius of the secondary source is *a*_3_ However, if *a*_3_ >|*M*|*a*_0_ the aperture does not (within the approximation we are using) block any of the light, therefore |*M*|*a*_0_ is the radius of the secondary source. The distinction between these two cases is necessary because when the small aperture is used *a*_3_ < |*M*|*a*_0_, whereas when no aperture is used *a_3_*>|*M*|*a*_0_.

The spectrum, *S*^(II)^(*ρ′, ω*), of the light in plane II can be calculated by using the result in Eq. (23):

$$\begin{array}{c} S^{\left(\mathrm{II}\right)}\left(\rho^{\prime },\omega \right)=W^{\left(\mathrm{II}\right)}\left(\rho^{\prime },\rho^{\prime },\omega \right),\\ =\frac{\pi a_{1}^{2}}{f_{2}^{2}}B_{0}\left(\omega \right){\left|t\left(\rho^{\prime }/f_{2},\omega \right)\right|}^{2}\text{circ}\left(\rho^{\prime }/a\right). \end{array}$$ (24)

We see from this expression that the secondary source occupies a circular domain of radius *a*, and that the spectrum of the light inside the secondary source is proportional to the product of spectral radiance in plane j, *B*_0_(*ω*), and transmittance of the interference filter, |*t*(*ρ′/f*_2_, *ω*)|^2^.

The complex degree of spectral coherence, $\mu^{\left(\mathrm{II}\right)}\left(\rho_{1}^{\prime },\rho_{2}^{\prime },\omega \right)$, of the light in the plane II is defined by the formula [16]:

$$\mu^{\left(\mathrm{II}\right)}\left(\rho_{1}^{\prime },\rho_{2}^{\prime },\omega \right)=\frac{W^{\left(\mathrm{II}\right)}\left(\rho_{1}^{\prime },\rho_{2}^{\prime },\omega \right)}{\sqrt{S^{\left(\mathrm{II}\right)}\left(\rho_{1}^{\prime },\omega \right)}\sqrt{S^{\left(\mathrm{II}\right)}\left(\rho_{2}^{\prime },\omega \right)}}.$$ (25)

Upon using Eqs. (23) and (24) in Eq. (25), we find that when $\rho_{1}^{\prime }<a$ and $\rho_{2}^{\prime }<a$, i.e., for pairs of points located inside the secondary source,

$$\begin{array}{c} \mu^{\left(\mathrm{II}\right)}\left(\rho_{1}^{\prime },\rho_{2}^{\prime },\omega \right)=E\left(\rho_{1}^{\prime },\rho_{2}^{\prime },\omega \right)\frac{t*\left(\rho_{1}^{\prime }/f_{2},\omega \right)}{\left|t\left(\rho_{1}^{\prime }/f_{2},\omega \right)\right|}\frac{t\left(\rho_{2}^{\prime }/f_{2},\omega \right)}{\left|t\left(\rho_{2}^{\prime }/f_{2},\omega \right)\right|}\\ \times \text{Besinc}\left(\frac{ka_{1}}{f_{2}}\left|\rho_{2}^{\prime }-\rho_{1}^{\prime }\right|\right). \end{array}$$ (26)

This result shows that *the group at NPL was incorrect in stating that the filter-lens combination eliminates the wavelength dependence of the complex degree of spectral coherence of the light in plane II*. In particular, since the first three factors on the right hand side of Eq. (26) are each unimodular, it follows that the modulus of $\mu^{\left(\mathrm{II}\right)}\left(\rho_{1}^{\prime },\rho_{2}^{\prime },\omega \right)$ is equal to the absolute value of Besinc $\left(ka_{1}\left|\rho_{2}^{\prime }-\rho_{1}^{\prime }\right|/f_{2}\right)$. Hence, the assertion by the group at NPL that the wavelength independence of $\mu^{\left(\mathrm{II}\right)}\left(\rho_{1}^{\prime },\rho_{2}^{\prime },\omega \right)$ causes the spectrum to change upon propagation from plane II to plane III is not correct. There may be a change of the spectrum (we will investigate that in the next section), but it is not caused by a wavelength independence of $\mu^{\left(\mathrm{II}\right)}\left(\rho_{1}^{\prime },\rho_{2}^{\prime },\omega \right)$.

In the next section we will refer to the effective correlation length, *L*(*ω*), of the light inside the secondary source. The correlation length of a Besinc type correlation function is typically taken as the smallest separation, $\left|\rho_{2}^{\prime }-\rho_{1}^{\prime }\right|$, for which the complex degree of spectral coherence takes on the value zero. Since Besinc has its first zero when its argument is equal to 3.832, it follows from Eqs. (26) and (3) that

$$L\left(\omega \right)=0.610\frac{\lambda f_{2}}{a_{1}}.$$ (27)

## 4. Spectrum of the Light On-Axis in Plane III

Let P be a point in plane III located by position vector *ρ*. When the Fresnel approximation is used to propagate the field from plane II to plane III, the spectrum of the light at P is given by the expression [17]

$$\begin{array}{c} S^{\left(\mathrm{III}\right)}\left(\rho ,\omega \right)=\frac{1}{{\left(\lambda z\right)}^{2}}\iint W^{\left(\mathrm{II}\right)}\left(\rho_{1}^{\prime },\rho_{2}^{\prime },\omega \right)\exp\left[ik\left(\rho_{2}^{\prime \prime 2}-\rho_{1}^{\prime \prime 2}\right)/2z\right]\\ \times \exp\left[-ik\left(\rho_{2}^{\prime }-\rho_{1}^{\prime }\right)\cdot \rho /z\right]d^{2}\rho_{1}^{\prime }d^{2}\rho_{2}^{\prime }, \end{array}$$ (28)

where $W^{\left(\mathrm{II}\right)}\left(\rho_{1}^{\prime },\rho_{2}^{\prime },\omega \right)$ is given by Eq. (23), and the integrations extend formally from − ∞ to ∞ in each variable.^4^

If the secondary source obeys the Leader condition [18]

$$\frac{kaL\left(\omega \right)}{z}\ll 1,$$ (29)

then we are in the far zone of the secondary source and the quadratic exponential term in Eq. (28) can be dropped. We will restrict our considerations to the on-axis observation point, *ρ* =0.

### 4.1 No Aperture in Plane II

In this case *a = |M |a*_0_, and the Leader condition is not fulfilled for the NPL parameter values. The on-axis spectrum is therefore given by the expression

$$\begin{array}{c} S^{\left(\mathrm{III}\right)}\left(0,\omega \right)=\frac{1}{{\left(\lambda z\right)}^{2}}\iint W^{\left(\mathrm{II}\right)}\left(\rho_{1}^{\prime },\rho_{2}^{\prime },\omega \right)\\ \times \exp\left[ik\left(\rho_{2}^{\prime \prime 2}-\rho_{1}^{\prime \prime 2}\right)/2z\right]d^{2}\rho_{1}^{\prime }d^{2}\rho_{2}^{\prime }, \end{array}$$ (30)

where $W^{\left(\mathrm{II}\right)}\left(\rho_{1}^{\prime },\rho_{2}^{\prime },\omega \right)$ is given by Eq. (23), with *a =|M|a*_0_.

In order to investigate *S*^(III)^(0, *ω*), let us first introduce average and difference vectors, $\bar{\rho }\prime$ and $\bar{\rho }\prime$, in plane II:

$$\bar{\rho }\prime =\frac{1}{2}\left(\rho_{2}^{\prime }+\rho_{1}^{\prime }\right),$$ (31a)

$$\bar{\rho }\prime =\rho_{2}^{\prime }-\rho_{1}^{\prime }.$$ (31b)

In terms of these variables, it follows from Eqs, (30), (23), (16) and (27) that

$$\begin{array}{c} S^{\left(\mathrm{III}\right)}\left(0,\omega \right)=\frac{\pi a_{1}^{2}}{\lambda^{2}z^{2}f_{2}^{2}}B_{0}\left(\omega \right)\iint G\left(\bar{\rho }\prime ,\bar{\rho }\prime ,\omega \right)\\ \times \exp\left(-ik\bar{\rho }\prime \cdot \bar{\rho }\prime /d\right)d^{2}\bar{\rho }\prime d^{2}\bar{\rho }\prime , \end{array}$$ (32)

where

$$\begin{array}{c} G\left(\bar{\rho }\prime ,\bar{\rho }\prime ,\omega \right)=t*\left(\left|\bar{\rho }\prime -\frac{1}{2}\bar{\rho }\prime \right|/f_{2},\omega \right)\\ \times t\left(\left|\bar{\rho }\prime +\frac{1}{2}\bar{\rho }\prime \right|/f_{2},\omega \right)\text{Besinc}\left[3.832\bar{\rho }\prime /L\left(\omega \right)\right]\\ \times \text{circ}\left[\left(\bar{\rho }\prime -\frac{1}{2}\bar{\rho }\prime \right)/\left|M\right|a_{0}\right]\text{circ}\left[\left(\bar{\rho }\prime +\frac{1}{2}\bar{\rho }\prime \right)/\left|M\right|a_{0}\right], \end{array}$$ (33)

and

$$\frac{1}{d}=\frac{1}{f_{2}}\left(\frac{D}{f_{2}}-1\right)-\frac{1}{z}.$$ (34)

Let us now examine the behavior of $G\left(\bar{\rho }\prime ,\bar{\rho }\prime ,\omega \right)$ as a function of the difference vector $\bar{\rho }\prime$ for a fixed value of the average vector $\bar{\rho }\prime$. The region over which the Besinc function is significant is the domain $\bar{\rho }\prime \leq L\left(\omega \right)$. As $\bar{\rho }\prime$ explores this region the first arguments of t* and *t* in Eq. (33) each change by at most the angle

$$\frac{L\left(\omega \right)}{f_{2}}=0.610\frac{\lambda }{a_{1}}.$$ (35)

For visible light this is an extremely small angle. For example, for λ =550 nm and *a*_1_ = 0.45 cm, *L*(*ω*)/*f*_2_ = 75 μrad. Therefore we will assume that the behavior of the interference filter is such that when evaluating the integral on the right hand side of Eq. (32) we may, to a good approximation, neglect the $\bar{\rho }\prime$ dependence of both t* and *t*, i.e., that we may approximate *G* as

$$\begin{array}{l} G\left(\bar{\rho }\prime ,\bar{\rho }\prime ,\omega \right)={\left|t\left(\bar{\rho }\prime /f_{2},\omega \right)\right|}^{2}\text{Besinc}\left[3.832\bar{\rho }\prime /L\left(\omega \right)\right]\\ \times \text{circ}\left[\left(\bar{\rho }\prime -\frac{1}{2}\bar{\rho }\prime \right)/\left|M\right|a_{0}\right]\text{circ}\left[\left(\bar{\rho }\prime +\frac{1}{2}\bar{\rho }\prime \right)/\left|M\right|a_{0}\right]. \end{array}$$ (36)

Furthermore, for the NPL system parameter values and the wavelengths used in their experiments, *L*(*ω*)≪|*M|a*_0_. Therefore we will now use the quasihomogeneous approximation [19] which corresponds to replacing Eq. (36) by the expression

$$G\left(\bar{\rho }\prime ,\bar{\rho }\prime ,\omega \right)={\left|t\left(\bar{\rho }\prime /f_{2},\omega \right)\right|}^{2}\text{Besinc}\left[3.832\bar{\rho }\prime /L\left(\omega \right)\right]\text{circ}\left(\bar{\rho }\prime /\left|M\right|a_{0}\right).$$ (37)

Upon substituting the approximate form Eq. (37) for *G* into Eq. (32), it can be shown, after some straightforward calculations, that the on-axis spectrum in plane III is given by the expression

$$S^{\left(\mathrm{III}\right)}\left(0,\omega \right)=\frac{\pi b^{2}}{z^{2}}B_{0}\left(\omega \right)M\left(\omega \right),$$ (38)

where

$$\begin{array}{c} M\left(\omega \right)=\frac{1}{\pi b^{2}}{\int \left|t\left(\bar{\rho }\prime /f_{2},\omega \right)\right|}^{2}\text{circ}\left(\bar{\rho }\prime /\left|M\right|a_{0}\right)\\ \times \text{circ}\left(\bar{\rho }\prime /b\right)d^{2}\bar{\rho }\prime , \end{array}$$ (39)

and *b = a*_1_*d*/*f*_2_. For the NPL parameter values, |*M*|*a*_0_ =0.48 cm and *b* =0.35 cm. Therefore, Eq. (39) simplifies to

$$\begin{array}{c} M\left(\omega \right)=\frac{1}{\pi b^{2}}\int {\left|t\left(\bar{\rho }\prime /f_{2},\omega \right)\right|}^{2}\text{circ}\left(\bar{\rho }\prime /b\right)d^{2}\bar{\rho }\prime ,\\ =\frac{2}{\beta^{2}}\int_{0}^{\beta }{\left|t\left(\theta ,\omega \right)\right|}^{2}\theta d\theta , \end{array}$$ (40)

where *β* = *b*/*f*_2_.

Let us now discuss the transmittance, |*t*(*θ*, *ω*)|^2^, of the interference filter. We will assume that it can be described by the Lissberger-Wilcock model [12–13]. This model works well for Fabry-Perot and all-dielectric interference filters for angles of incidence which are less than 20°. It can also be used to describe, less accurately, double halfwave and induced transmission filters [20].

Let λ_0_ denote the peak wavelength transmitted by the interference filter at normal incidence (*θ* = 0), (Δλ)_0_ denote its bandwidth (FWHM) at normal incidence, and *T*_0_ denote its maximum transmittance. According to the Lissberger-Wilcock model the transmittance of the interference filter is given by the expression

$${\left|t\left(\theta ,\omega \right)\right|}^{2}=T_{0}{\left\{1+{\left[\frac{2\left(\lambda -\lambda_{0}\right)}{{\left(\Delta \lambda \right)}_{0}}+\frac{\lambda_{0}}{{\left(\Delta \lambda \right)}_{0}}\frac{\theta^{2}}{\eta^{2}}\right]}^{2}\right\}}^{-1},$$ (41)

where *η* is the effective index of refraction of the interference filter.

The integral in Eq. (40) can be evaluated (see Ref. [12]) without any approximation, for the transmittance function Eq. (41). The result is that

$$M\left(\omega \right)=\frac{T_{0}}{2X}\tan^{-1}\left[\frac{2X}{1+\xi \left(\xi +2X\right)}\right],$$ (42)

where *X* = λ_0_*β*^2^/[2*η*^2^(Δλ)_0_], and *ξ* = 2(λ − λ_0_)/(Δλ)_0_.

Some comments about the interference filter parameters λ_0_, (Δλ)_0_, and η are now in order. The effective indices of refraction, i.e., the values of η, for the filters used in the NPL experiments are not known. However, typical values of η are in the range 1.4<η<3.4, with the lower values being more typical [21–25]. We will therefore use η = 2 in our calculations. The precise values of λ_0_ and (Δλ)_0_ are also not known. For reasons which will be explained in Sec. 4.3, we will take the values of λ_0_ for the six filters to be 421.9 nm, 484.0 nm, 512.4 nm, 566.0 nm, 609.1 nm, and 652.0 nm, with their band-widths being, respectively, 9 nm, 9 nm, 5 nm, 13 nm, 8 nm, and 8 nm.

### 4.2 Small Aperture in Plane II

In this case *a*_3_ = 0.012 cm and the Leader condition is fulfilled. It therefore follows from Eqs. (28) and (23) that the spectrum can be written as

$$\begin{array}{c} S^{\left(\mathrm{III}\right)}\left(0,\omega \right)=\frac{\pi a_{1}^{2}}{\lambda^{2}z^{2}f_{2}^{2}}B_{0}\left(\omega \right)\int_{A}\int_{A}t*\left(\rho_{1}^{\prime }/f_{2},\omega \right)t\left(\rho_{2}^{\prime }/f_{2},\omega \right)\\ \times E\left(\rho_{1}^{\prime },\rho_{2}^{\prime },\omega \right)\text{Besinc}\left(\frac{ka_{1}}{f_{2}}\left|\rho_{2}^{\prime }-\rho_{1}^{\prime }\right|\right)d^{2}\rho_{1}^{\prime }d^{2}\rho_{2}^{\prime }, \end{array}$$ (43)

where A is the circular aperture of radius *a*_3_. For the same reasons as were discussed in the Sec. 4.1, we will assume that the product of t* and *t* on the right hand side of Eq. (43) can be replaced by |*t*|^2^ evaluated at the average position, i.e., that to a good approximation,

$$\begin{array}{c} S^{\left(\mathrm{III}\right)}\left(0,\omega \right)=\frac{\pi a_{1}^{2}}{\lambda^{2}z^{2}f_{2}^{2}}B_{0}\left(\omega \right)\int_{A}\int_{A}{\left|t\left(\left|\rho_{1}^{\prime }+\rho_{2}^{\prime }\right|/2f_{2},\omega \right)\right|}^{2}E\left(\rho_{1}^{\prime },\rho_{2}^{\prime },\omega \right)\\ \times \text{Besinc}\left(\frac{ka_{1}}{f_{2}}\left|\rho_{2}^{\prime }-\rho_{1}^{\prime }\right|\right)d^{2}\rho_{1}^{\prime }d^{2}\rho_{2}^{\prime }, \end{array}$$ (44)

For the NPL parameter values, it can be shown from Eq. (41) that, to a very good approximation, ${\left|t\left(\left|\rho_{2}^{\prime }+\rho_{1}^{\prime }\right|/2f_{2},\omega \right)\right|}^{2}\approx {\left|t\left(0,\omega \right)\right|}^{2}$, for all $\rho_{1}^{\prime }$ and $\rho_{2}^{\prime }$ which are in A. The on-axis spectrum in plane III is therefore given, to a good approximation, by the expression

$$S^{\left(\mathrm{III}\right)}\left(0,\omega \right)=\frac{\pi a_{3}^{2}}{z^{2}}B_{0}\left(\omega \right){\left|t\left(0,\omega \right)\right|}^{2}N\left(\omega \right),$$ (45)

where

$$\begin{array}{l} N\left(\omega \right)=\frac{a_{1}^{2}}{\lambda^{2}a_{3}^{2}f_{2}^{2}}\int_{A}\int_{A}E\left(\rho_{1}^{\prime },\rho_{2}^{\prime },\omega \right)\\ \times \text{Besinc}\left(\frac{ka_{1}}{f_{2}}\left|\rho_{2}^{\prime }-\rho_{1}^{\prime }\right|\right)d^{2}\rho_{1}^{\prime }d^{2}\rho_{2}^{\prime }. \end{array}$$ (46)

This integral can be evaluated by substituting the Fourier integral representation of the Besinc function [see Eq. (88)] into the integral, interchanging the orders of integration, and then recognizing the resultant integrals as familiar diffraction integrals. The result is that

$$N\left(\omega \right)=1-\sum_{s=0}^{\propto }\frac{(-1{)}^{s}}{2s+1}(\frac{u}{v}{)}^{2s}Q_{2s}\left(v\right),$$ (47)

where

$$u=\frac{ka_{3}^{2}}{f_{2}}\left(\frac{D}{f_{2}}-1\right),$$ (48)

$$v=\frac{ka_{3}a_{1}}{f_{2}},$$ (49)

and the *Q*_2t_(*v*) functions are those introduced by Wolf [26] and simplified by Petersen [27]:

$$Q_{0}\left(v\right)=J_{0}^{2}\left(v\right)+J_{1}^{2}\left(v\right),$$ (50)

$$Q_{2s}\left(v\right)=-\frac{2s+1}{2s\left(s+1\right)}\left\{v\left[J_{2}\left(v\right)J_{2s+1}\left(v\right)-J_{1}\left(v\right)J_{2s+2}\left(v\right)\right]+2sJ_{1}\left(v\right)J_{2s+1}\left(v\right)\right\},\;\text{if }s>0.$$ (51)

### 4.3 Numerical Investigation of the Spectrum On-Axis in Plane III; Comparison to the NPL Experimental Values

Let $s_{N}^{\left(\mathrm{III}\right)}(0,\lambda )$ be the spectrum, as a function of wavelength, at the on-axis position in plane III, for the case in which there is no aperture in plane II. Let the wavelength at which this spectrum peaks be denoted by λ_P_ and the bandwidth (FWHM) of this spectrum be denoted by Δλ. Let $s_{\lambda }^{\left(\mathrm{III}\right)}(0,\lambda )$ be the spectrum, as a function of wavelength, at the on-axis position in plane III, for the case where the small aperture is in plane II. Let $\lambda_{P}^{\prime }$ denote the peak wavelength for this spectrum. The group at NPL measured λ_P_, Δλ, and $\lambda_{P}^{\prime }$ for six different interference filters [1]. Their results are shown in Table 1, along with the shift,

$$\delta \lambda_{P}=\lambda_{P}^{\prime }-\lambda_{P},$$ (52)

which occurred due to the insertion of the aperture.

There are several things which should be noticed about the results shown in Table 1. First, the shifts are, in absolute value, of the order of 0.5 nm to 2.0 nm. Secondly, some of the shifts are blueshifts and some are redshifts. Furthermore, there are no obvious trends in the behaviors of the shifts as functions of either λ_P_ or Δλ.

Let us now investigate the shifts that our theory predicts. It follows from Eqs. (38) and (42) that the spectrum when there is no aperture in plane II is given by the expression

$$s_{N}^{\left(\mathrm{III}\right)}(0,\lambda )=\frac{\pi b^{2}}{z^{2}}b_{0}(\lambda )m_{N}(\lambda ),$$ (53)

where *b*_0_(λ) is the spectral radiance, as a function of wavelength, at the exit aperture of the integrating sphere and

$$m_{N}(\lambda )=\frac{T_{0}}{2X}\tan^{-1}\left[\frac{2X}{1+\xi (\xi +2X)}\right].$$ (54)

Here *ξ* and *X* are given by the expressions which follow Eq. (42). It follows from Eqs. (45), (47), and (41) that the spectrum when the small aperture is in plane II is given by the expression

$$s_{A}^{\left(\mathrm{III}\right)}(0,\lambda )=\frac{\pi a_{1}^{2}}{z^{2}}b_{0}(\lambda )m_{A}(\lambda ),$$ (55)

where

$$m_{A}(\lambda )=T_{0}N(2\pi c/\lambda ){\left\{1+{\left[\frac{2(\lambda -\lambda_{0})}{{\left(\Delta \lambda \right)}_{0}}\right]}^{2}\right\}}^{-1}.$$ (56)

In order to simulate the experimental conditions, we did the following things. The color temperature of the lamp was 3200 K [7]. We therefore took the spectral radiance, *b*_0_(λ), to be a Planck distribution [28]:

$$b_{0}(\lambda )=\frac{2hc^{2}}{\lambda^{5}}\frac{1}{\exp(hc/\lambda k_{B}T)-1},$$ (57)

with a temperature of *T* = 3200 K. In Eq. (57)*h* is Planck’s constant and *k*_B_ is Boltzmann’s constant. Furthermore, we chose the center frequencies, λ_0_, and the bandwidths, (Δλ)_0_, of the interference filters to be such that the λ_P_ and Δλ values obtained for the spectrum $s_{N}^{\left(\mathrm{III}\right)}(0,\lambda )$ [from Eq. (53)] agreed with the experimental values of Table 1 to the number of decimal places being displayed there.

Table 2 lists the interference filter parameters, and λ_0_ and (Δλ)_0_, we used in our calculations and gives the shifts, δλ_P_, predicted by our theory. These shifts were obtained by using a search routine to find $\lambda_{P}^{\prime }$ and λ_P_ from, respectively, Eqs. (55) and (53), and then subtracting the two values.

These shifts disagree with those measured by the group at NPL. The theoretical shifts are approximately two orders of magnitude smaller than the experimental shifts. Furthermore, the theoretical shifts are always redshifts, whereas the experimental shifts are in some cases redshifts and in other cases blueshifts,

At this point some further comments about the shifts predicted by our theory are in order. First, let us consider $\lambda_{P}^{\prime }$. $s_{A}^{\left(\mathrm{III}\right)}(0,\lambda )$ is the product of two wavelength dependent factors, *m*_A_(λ) and *b*_0_(λ). Due to the presence of the factor *N*(2*πc*/λ), *m*_A_(λ) is not centered at λ_0_, it is blueshifted from it by an amount of the order of 0.0001 nm. Multiplication by the Planck spectrum then causes *m*_A_(λ) to be redshifted by about 0.1 nm. Now, let us consider λ_P_. $s_{N}^{\left(\mathrm{III}\right)}(0,\lambda )$ is the product of two wavelength dependent factors, *m*_N_(λ) and *b*_0_(λ). *m*_N_(λ) is not centered at λ_0_, it is blueshifted from this value by an amount of the order of 0.01 run. Multiplication by the Planck spectrum then causes *m*_N_(λ) to be redshifted by about 0.1 nm. When we subtract λ_P_ from $\lambda_{P}^{\prime }$ the two Planck shifts cancel. Since the blueshift of *m*_N_(λ) is much larger than the blueshift of *m*_A_(λ), the resulting shift, δλ_P_, is a redshift of the order of 0.01 nm.

The physical origin of this redshift is as follows. The field incident upon the interference filter can be represented as a superposition of polychromatic plane waves traveling in different directions, and the wavelength at which the transmittance of the interference filter peaks decreases as the angle of incidence of a plane wave increases [see Eq. (41)]. Therefore, for each polychromatic plane wave incident upon the interference filter at an oblique angle of incidence, the spectrum of the transmitted light has a peak wavelength which is less than it would be for a normally incident plane wave. In the case in which no aperture was used, the contributions from a significant set of such waves arrive at the on-axis observation point in plane III. However, in the case in which the small aperture was used, the contributions from a large subset of these waves were blocked at plane II and did not arrive at plane III. As a result, the spectrum in plane III when the small aperture is used peaks at a longer wavelength than it does when no aperture is used.

## 5. Conclusion

Our paper contains two separate sets of results. (1) In Sec. 3 an approximate form for the cross-spectral density of the light in plane II was obtained [Eq. (23)], and it was shown that the corresponding complex degree of spectral coherence contradicts the explanation for the shifts given in Ref. [1]. (2) In Sec. 4 further approximations were made to propagate the cross-spectral density obtained in Sec. 3 from plane II to plane III, and an approximate expression for the on-axis spectrum was obtained, both for the case in which no aperture is used, and for the case in which the small aperture is used. It was found that the peak wavelength of the spectrum in the latter case is shifted with respect to the peak wavelength for the former case. However the shifts predicted by our analysis are much smaller than those reported in Ref. [1], so small as to be unobservable to within the accuracy of their experiments.

This brings us to an important question. Our analysis is predicting no observable shift (to within the accuracy of the measurements of the group at NPL), and yet the group at NPL observed shifts; so where is this shift coming from? In our opinion there are three possibilities. One possibility is that their interference filters did not behave in the manner we have assumed in our calculations, and that shifts arose as a result. A second possibility has to do with the spatial coherence properties of the light in plane III. Since the secondary source created in plane II when no aperture is used has a radius which is forty times as large as in the case in which the small aperture is used, the spatial coherence properties of the light in plane III will be quite different in the two cases. If this difference is significant enough, it may be that the monochromator used in the detection process responds differently in the two cases. A third possibility, and in our opinion the best one, is that the shift is caused by chromatic aberration introduced by L_1_, or L_2_, or both. L_1_ has a short focal length and is not an achromatic doublet, and is therefore an obvious candidate for introducing significant chromatic aberration. L_2_ is an achromatic doublet. However, it can only be perfectly achromatic for two wavelengths, whereas filters with six quite different center wavelengths were used in the NPL experiments.

Finally, let us comment on the relevance of these possibilities for typical spectral irradiance measurement systems. Typical spectral irradiance measurement systems do not use interference filters. Therefore, if the shift is caused by a nonideal behavior of the interference filter, it is irrelevant for typical spectral irradiance measurements. As concerns the second possibility, it has been known for some time that the spatial coherence properties of the light incident upon a monochromator do effect its response, viz., its slit scattering function [29–30]. Hence any comparison of experimentally measured spectra which have significantly different spatial coherence properties at the entrance aperture of the monochromator should take this into account. Finally, if the shift is caused by chromatic aberration, it will not occur in spectral irradiance systems which use only mirrors, and is an effect which should obviously be taken into account in any system which uses lenses.

## Figures and Tables

**Table 1:** NPL experimental results

λ_P_, in nm	Δλ, in nm	$\lambda_{P}^{\prime \prime }$, in nm	δλ_P_, in nm
422.0	9	421.0	−1.0
484.1	9	483.6	−0.5
512.4	5	514.1	1.7
566.1	13	564.1	−2.0
609.1	8	610.3	1.2
652.0	8	653.2	1.2

**Table 2:** Theoretical values for the shift

λ_0_, in nm	(Δλ)_0_, in nm	δλ_P_, in nm
421.9	9	0.007
484.0	9	0.009
512.4	5	0.009
566.0	13	0.010
609.1	8	0.011
652.0	8	0.012

## Footnotes

1 In Ref. [1] the diameter of the smaller aperture was incorrectly reported as 0.24 cm. The correct value is 0.024 cm [8].

2 In order for Eq. (2) to be appropriate, the radius of the source must be much larger than the wavelength of the light. In the NPL experiments the wavelengths of interest are visible and the radius of the exit aperture of the integrating sphere is 0.12 cm, so this condition is fulfilled.

3 The spectrum *S* is also sometimes referred to as the spectral density of the light. It is the optical intensity per unit angular frequency at the position and angular frequency in question [15].

4 In practice, the circ functions in Eq. (23) limit the domains of integration to the area occupied by the secondary source.

## 6. Appendix A: Coherent Impulse Response Function for the Propagation from Plane I to Plane II

### 6.1 Field Exiting L_1_

Let Q be a point in the plane occupied by L_1_, and let *α*_1_ be the two-dimensional position vector in the plane which locates Q. The field, *U*^(I−)^(*α*_1,_ *ω*), incident upon L_1_ at the point Q due to a monochromatic, unit amplitude, point source at position P″ in plane I is given by the expression

$$U^{\left(1-\right)}(\alpha_{1},\omega )=\frac{1}{i\lambda }\frac{\exp(ik|r_{1}-\rho^{\prime \prime }|)}{\left|r_{1}-\rho^{\prime \prime }\right|},$$ (58)

where *r*_1_ = (*α*_1_, *f*_1_). Since the angles involved are small, this expression may be approximated (paraxial approximation) as

$$U^{\left(1-\right)}(\alpha_{1},\omega )=\frac{\exp(ikf_{1})}{i\lambda f_{1}}\exp[ik{\left(\alpha_{1}-\rho^{\prime \prime }\right)}^{2}/2f_{1}].$$ (59)

The field exiting the lens, *U*^(I+)^(*α*_1,_ *ω*), is given by the expression [31]

$$\begin{array}{c} U^{\left(1+\right)}(\alpha_{1},\omega )=\\ U^{\left(1-\right)}(\alpha_{1},\omega )\exp(-ik\alpha_{1}^{2}/2f_{1})P_{1}(\alpha_{1}), \end{array}$$ (60)

where *P*(*α*_1_) is the pupil function for *L*_1_, *P*_1_(*α*_1_) =circ(*α*_1_/*a*_1_). Using Eq. (59) in Eq. (60) yields

$$\begin{array}{c} U^{\left(1+\right)}(\alpha_{1},\omega )=\\ \frac{\exp[ik\phi (\rho^{\prime \prime })]}{i\lambda f_{1}}\exp(-ik\alpha_{1}\cdot \rho^{\prime \prime }/f_{1})P_{1}(\alpha_{1}), \end{array}$$ (61)

where

$$\phi (\rho^{\prime \prime })=f_{1}+(\rho^{\prime \prime 2}/2f_{1}).$$ (62)

In the next subsection we will need ${\bar{U}}^{\left(1+\right)}(K,\omega )$, the two-dimensional spatial Fourier transform of *U*^(I+)^(*α*_1_, *ω*),

$$\begin{array}{c} {\bar{U}}^{\left(1+\right)}(K,\omega )=\\ \int U^{\left(1+\right)}(\alpha_{1},\omega )\exp(-iK\cdot \alpha_{1})d^{2}\alpha_{1}, \end{array}$$ (63)

evaluated at *K = ks*_⊥_. It follows from Eqs. (61) and (63) that

$$\begin{array}{c} {\bar{U}}^{\left(1+\right)}(ks_{⊥},\omega )=\frac{\exp[ik\phi (\rho^{\prime \prime })]}{i\lambda f_{1}}{\bar{P}}_{1}[k(s_{⊥}-s_{0⊥})],\\ \;=\frac{\pi a_{1}^{2}}{i\lambda f_{1}}\exp[ik\phi (\rho^{\prime \prime })]\\ \;\times \text{Besinc}[ka_{1}|s_{⊥}-s_{0⊥}|], \end{array}$$ (64)

where *s*_0⊥_ = −*ρ″/f*_1_

### 6.2 Field Incident Upon L_2_

Let P be a point in-between L_1_ and the interference filter. Let *r* be a position vector which goes from the center of L_1_ to P. The field at P can be represented as a superposition (angular spectrum) of plane waves [32]:

$$U(r,\omega )=\int a(s_{⊥},\omega )\exp(iks\cdot r)d^{2}s_{⊥}.$$ (65)

where *s* = (s_⊥_, *s_z_*) is a unit vector,

$$\begin{array}{c} s_{⊥}=\sqrt{1-s_{⊥}^{2}},\;\text{if}\;s_{⊥}^{2}\leq 1,\\ \;=i\sqrt{s_{⊥}^{2}-1},\;\text{if}\;s_{⊥}^{2}>1, \end{array}$$ (66)

and

$$a(s_{⊥},\omega )=\frac{1}{\lambda^{2}}{\bar{U}}^{\left(1+\right)}(ks_{⊥},\omega ).$$ (67)

In Eq. (66), *s*_⊥_ = |*s*_⊥_|.

In spherical polar coordinates, as measured from the center of *L*_1_, *s* = (l, *θ, ϕ*). In this notation s_⊥_ = (*s_z_, s_y_*) = (sin*θ*cos*ϕ*, sin*θ*sin*ϕ*). Therefore the angle of incidence of the plane wave exp(iks • r) with respect to the normal to the surface of the IF is *θ* [see Fig. 2], where s_⊥_=sin *θ ≈ θ.*

Let *t*(*θ*, *ω*) be the amplitude transmission function of the filter for a plane wave of angular frequency *ω* and angle of incidence *θ.* If we neglect the finiteness of the transverse size of the IF, the effect of the interference filter is to change the amplitude of each plane wave, exp(iks • r), by the factor *t*(s_⊥_, *ω*). The amplitude, call it $\hat{a}\left(s_{⊥},\omega \right)$, of the plane wave exiting the filter is therefore

$$\begin{array}{l} \hat{a}(s_{⊥},\omega )=t(s_{⊥},\omega )a(s_{⊥},\omega ),\\ \;=\frac{1}{\lambda^{2}}t(s_{⊥},\omega ){\bar{U}}^{\left(1+\right)}(ks_{⊥},\omega ),\\ \;=\frac{\pi a_{1}^{2}}{i\lambda^{3}f_{1}}t(s_{⊥},\omega )\exp[ik\phi (\rho^{\prime \prime })]\\ \;\times \text{Besinc}[ka_{1}|s_{⊥}-s_{0⊥}|]. \end{array}$$ (68)

Let *r*_2_ = (*α*_2_ *D*) be the position vector which goes from the center of L_1_ to the point in L_2_ located by the two-dimensional position vector *α*_2_. The field, *U*^(2−)^(*α*_2,_ *ω*) incident on L_2_ can be represented as a superposition of plane waves:

$$\begin{array}{l} U^{\left(2-\right)}(\alpha_{2},\omega )=\int \hat{a}(s_{⊥},\omega )\exp(iks\cdot r)d^{2}s_{⊥},\\ \;=\int \hat{a}(s_{⊥},\omega )\exp(iks_{⊥}\cdot \alpha_{2})\\ \;\times \exp(iks_{z}D)d^{2}s_{⊥}. \end{array}$$ (69)

Since the angles involved are small, we may make the approximation. $s_{z}=\sqrt{1-s_{⊥}^{2}}\approx 1-\frac{1}{2}s_{⊥}^{2}$ in Eq. (69), and we find that

$$\begin{array}{c} U^{\left(2-\right)}(\alpha_{2},\omega )=\exp(ikD)\int \hat{a}(s_{1},\omega )\exp(iks_{⊥}\cdot \alpha_{2})\\ \times \exp(-ikDs_{⊥}^{2}/2)d^{2}s_{⊥}. \end{array}$$ (70)

### 6.3 Final Expression

The coherent impulse response, *h*(*ρ*′, *ρ*″, *ω*), for the propagation from plane T to plane II is the field in plane II due to the point source in plane I, i.e.,

$$h(\rho^{\prime },\rho^{\prime \prime },\omega )=U^{\left(\mathrm{II}\right)}(\rho^{\prime },\omega ).$$ (71)

If we neglect the finite transverse size of L_2_, this equation can be written as [33]

$$h(\rho^{\prime },\rho^{\prime \prime },\omega )=\frac{\exp[ik\psi (\rho^{\prime })]}{i\lambda f_{2}}{\bar{U}}^{\left(2-\right)}(k\rho^{\prime }/f_{2},\omega ),$$ (72)

where *ψ*(*ρ*′) = *f*_2_+(*ρ*′^2^/2*f*_2_). It follows from Eq. (70) that

$$\begin{array}{c} {\bar{U}}^{\left(2-\right)}(ks_{⊥},\omega )=\\ \lambda^{2}\hat{a}(s_{⊥},\omega )\exp(ikD)\exp(-ikDs_{⊥}^{2}/2). \end{array}$$ (73)

Upon substituting Eq. (73) in Eq. (72) and Eq. (68) in the result we find that

$$\begin{array}{c} \begin{array}{c} h(\rho^{\prime },\rho^{\prime \prime },\omega )=\\ -\frac{\pi a_{1}^{2}}{\lambda^{2}f_{1}f_{2}}\exp\left\{ik[\phi (\rho^{\prime \prime })+\chi (\rho^{\prime })]\right\} \end{array}\\ \times \;\text{Besinc}\;\text{(}ka_{1}|(\rho \prime \;/f_{2})\;-\;(\rho "\;/f_{1})|)\;t\;(\rho \prime \;/f_{2,}\;\omega ), \end{array}$$ (74)

where

$$\chi (\rho^{\prime })=f_{2}+D+\left(1-\frac{D}{f_{2}}\right)({\rho^{\prime }}^{2}/2f_{2}).$$ (75)

The magnification of the imaging from plane I to plane II is *M = −f*_2_/*f*_1_. It therefore follows from Eq. (74) that the coherent impulse response function can be written as

$$h(\rho^{\prime },\rho^{\prime \prime },\omega )=t(\rho^{\prime }/f_{2},\omega )h_{N}(\rho^{\prime },\rho^{\prime \prime },\omega ),$$ (76)

where *h*_N_(*ρ*′, *ρ*″, *ω*) is the coherent impulse response function if there is no interference filter,

$$\begin{array}{c} h_{N}(\rho^{\prime },\rho^{\prime \prime },\omega )=C(\omega )\exp\{ik[\phi (\rho^{\prime \prime })+\chi (\rho^{\prime })]\}\\ \times \text{Besinc}\left(\frac{ka_{1}}{f_{1}}\left|\rho^{\prime \prime }-\frac{1}{M}\rho^{\prime }\right|\right). \end{array}$$ (77)

Here *ϕ*(*ρ*″) and χ(*ρ*′) are given by Eqs. (62) and (75) respectively, and

$$C(\omega )=\frac{1}{M}\frac{\pi a_{1}^{2}}{\lambda^{2}f_{1}^{2}}.$$ (78)

## 7. Appendix B: Derivation of $\bar{E}\left(\rho_{1}^{\prime \prime },\rho_{2}^{\prime \prime },\omega \right)\approx 1$

Let us first define average and difference variables in plane I as, respectively,

$${\bar{\rho }}^{\prime \prime }=\frac{1}{2}(\rho_{2}^{\prime \prime }+\rho_{1}^{\prime \prime }),$$ (79a)

$${\bar{\rho }}^{\prime \prime }=\rho_{2}^{\prime \prime }-\rho_{1}^{\prime \prime }.$$ (79a)

In terms of these variables $\bar{E}\left(\rho_{1}^{\prime \prime },\rho_{2}^{\prime \prime },\omega \right)$ [see Eq. (17)] can be rewritten as

$$\bar{E}(\rho_{1}^{\prime \prime },\rho_{2}^{\prime \prime },\omega )=\exp\left(\frac{2\pi i}{\lambda f_{1}}{\bar{\rho }}^{\prime \prime }\cdot {\bar{\rho }}^{\prime \prime }\right).$$ (80)

As $\rho_{1}^{\prime \prime }$ and $\rho_{2}^{\prime \prime }$ each explore the domain σ, the maximum value of ${\bar{\rho }}^{\prime \prime }=\left|{\bar{\rho }}^{\prime \prime }\right|$ is *a*_0_. in the integrand of Eq. (15) the range of values of ${\bar{\rho }}^{\prime \prime }=\rho_{2}^{\prime \prime }-\rho_{1}^{\prime \prime }$ over which *j*_0_ is significant is ${\bar{\rho }}^{\prime \prime }\leq \lambda /2$. Hence as $\rho_{1}^{\prime \prime }$ and $\rho_{2}^{\prime \prime }$ range over the domain where the integrand is significant,

$$\left|\frac{2\pi }{\lambda f_{1}}{\bar{\rho }}^{\prime \prime }\cdot {\bar{\rho }}^{\prime \prime }\right|\leq \frac{\pi a_{0}}{f_{1}}.$$ (81)

For the NPL system *a*_0_ = 0.12 cm and *f*_1_ = 5 cm; therefore over the domain where the integrand is significant the real and imaginary parts of $\bar{E}\left(\rho_{1}^{\prime \prime },\rho_{2}^{\prime \prime },\omega \right)$ fulfill, respectively, the inequalities:

0.997≤Re[E¯(ρ1″,ρ2″,ω)]≤1 (82a)

−0.075≤Im[E¯(ρ1″,ρ2″,ω)]≤0.075 (82b)

## 8. Appendix C: Derivation of Eq. (19)

Let

$$\begin{array}{c} B(\rho_{1}^{\prime },\rho_{2}^{\prime },\omega )=\int_{\sigma }\int_{\sigma }\text{Besinc}\left(\frac{ka_{1}}{f_{1}}\left|\rho_{1}^{\prime \prime }-\frac{1}{M}\rho_{1}^{\prime }\right|\right)\\ \times \text{Besinc}\left(\frac{ka_{1}}{f_{1}}\left|\rho_{2}^{\prime \prime }-\frac{1}{M}\rho_{2}^{\prime }\right|\right)j_{0}(k|\rho_{2}^{\prime \prime }-\rho_{1}^{\prime \prime }|)d^{2}\rho_{1}^{\prime \prime }d^{2}\rho_{2}^{\prime \prime }, \end{array}$$ (83)

and let us make the change of variables

$$u_{j}=\rho_{j}^{\prime \prime }/a_{0},\;(j=1,2)$$ (84a)

$$v_{j}=\rho_{j}^{\prime }/Ma_{0},\;(j=1,2)$$ (84b)

In terms of these new variables, Eq. (83) can be rewritten as

$$\begin{array}{c} B(\rho_{1}^{\prime },\rho_{2}^{\prime },\omega )=a_{0}^{4}\int_{u.d.}\int_{u.d.}\text{Besinc}(\kappa_{1}|u_{1}-v_{1}|)\\ \times \text{Besinc}(\kappa_{1}|u_{2}-v_{2}|)j_{0}(\kappa |u_{2}-u_{1}|)d^{2}u_{1}d^{2}u_{2}, \end{array}$$ (85)

where *u.d.* is the unit disk, *u.d.* ={*u*:*u*≤1}, and

$$\kappa_{1}=\frac{ka_{1}a_{0}}{f_{1}},$$ (86a)

$$\kappa =ka_{0}.$$ (86b)

If *κ*_I_ ≫ 1 then the first and second Besinc functions in the integrand on the right hand side of Eq. (85) are, respectively, very sharply peaked about the values $\mu_{1}=\nu_{1}=\rho_{1}^{\prime }/Ma_{0}$ and $\mu_{2}=\nu_{2}=\rho_{2}^{\prime }/Ma_{0}$. As a result, if *v*_1_ or *v*_2_ lies outside the unit disk, then the integral on the right hand side of Eq. (85) is approximately zero, and if *v*_1_ and *v*_2_ both lie within the unit disk the integrals in Eq. (85) may be approximated by extending the limits to ± ∞, i.e.,

$$\begin{array}{c} B(\rho_{1}^{\prime },\rho_{2}^{\prime },\omega )\approx a_{0}^{4}\text{circ}(\rho_{1}^{\prime }/Ma_{0})\text{circ}(\rho_{2}^{\prime }/Ma_{0})\\ \times \int \int \text{Besinc}(\kappa_{1}|u_{1}-v_{1}|)\;\text{Besinc}(\kappa_{1}|u_{2}-v_{2}|)\\ \times j_{0}(\kappa |u_{2}-u_{1}|)d^{2}u_{1}d^{2}u_{2}. \end{array}$$ (87)

The right hand side of Eq. (87) may be simplified by using the Fourier representations of the functions in the integrand:

$$\begin{array}{l} \text{Besinc}(\kappa_{1}|u_{j}-v_{j}|)=\frac{1}{\pi \kappa_{1}^{2}}\int \text{circ}(K_{j}/\kappa_{1})\\ \times \exp[-iK_{j}\cdot (u_{j}-v_{j})]d^{2}K_{j},(j=1,2), \end{array}$$ (88)

$$\begin{array}{c} j_{0}(\kappa |u_{2}-u_{1}|)=\\ \frac{1}{2\pi \kappa^{2}}\int \frac{\text{circ}(K/\kappa )}{\sqrt{1-{\left(K/\kappa \right)}^{2}}}\exp[-iK\cdot (u_{2}-u_{1})]d^{2}K. \end{array}$$ (89)

Upon substituting Eqs. (88) and (89) into Eq. (87), we find, after performing some simple integrations, that

$$\begin{array}{c} B(\rho_{1}^{\prime },\rho_{2}^{\prime },\omega )=D(\omega )\text{circ}(\rho_{1}^{\prime }/Ma_{0})\text{circ}(\rho_{2}^{\prime }/Ma_{0})\\ \times \int \frac{\text{circ}(K/\kappa_{1})\text{circ}(K/\kappa )}{\sqrt{1-{\left(K/\kappa \right)}^{2}}}\exp[iK\cdot (v_{2}-v_{1})d^{2}K,\\ =D(\omega )\text{circ}(\rho_{1}^{\prime }/Ma_{0})\text{circ}(\rho_{2}^{\prime }/Ma_{0})\int \frac{\text{circ}(K/\kappa_{1})}{\sqrt{1-{\left(K/\kappa \right)}^{2}}}\\ \times \exp[iK\cdot (v_{2}-v_{1})]d^{2}K, \end{array}$$ (90)

where

$$D(\omega )=\frac{{\left(2\pi a_{0}\right)}^{4}}{{\left(\pi \kappa_{1}^{2}\right)}^{2}2\pi \kappa^{2}},$$ (91)

and the step performed in Eq. (90) follows from the fact that *κ*_1_/*κ = a*_1_/*f*_1_ < 1.

If (*a*_1_/*f*_1_)^2^≪ 1, Eq. (90) can be approximated as

$$\begin{array}{c} B\left(\rho_{1}^{\prime },\rho_{2}^{\prime },\omega \right)=D\left(\omega \right)\text{circ}\left(\rho_{1}^{\prime }/Ma_{0}\right)\text{circ}\left(\rho_{2}^{\prime }/Ma_{0}\right)\\ \times \int \text{circ}\left(K/\kappa_{1}\right)\exp[iK\cdot \left(v_{2}-v_{1}\right)d^{2}K,\\ =D\left(\omega \right)\text{circ}\left(\rho_{1}^{\prime }/Ma_{0}\right)\text{circ}\left(\rho_{2}^{\prime }/Ma_{0}\right)\pi \kappa_{1}^{2}\\ \times \text{Besinc}\left(\kappa_{1}\left|v_{2}-v_{1}\right|\right),\\ =\frac{1}{2C^{2}\left(\omega \right)}{\left(\frac{a_{1}}{f_{2}}\right)}^{2}\text{Besinc}\left(\frac{ka_{1}}{f_{2}}\left|\rho_{2}^{\prime }-\rho_{1}^{\prime }\right|\right)\text{circ}\left(\rho_{1}^{\prime }/|M|a_{0}\right)\times \text{circ}\left(\rho_{2}^{\prime }/|M|a_{0}\right). \end{array}$$ (92)

For the NPL system, *a*_0_ = 0.12 cm and *a*_1_/*f*_1_ = 0.09, and the peak wavelengths of the interference Filters used in their experiments were in the range 422 nm to 652 nm. For these parameter values, $1.04\times 10^{3}\leq \frac{ka_{1}a_{0}}{f_{1}}\leq 1.61\times 10^{3}$ and (*a*_1_/*f*_1_)^2^ = 8.10× 10^−3^; therefore, *κ*_1_ ≫ 1 and (*a*_1_/*f*_1_)^2^≪ 1. It then follows that the approximation Eq. (92) is appropriate for the NPL system.
